# Reemergence of St. Louis Encephalitis Virus, California, 2015

**DOI:** 10.3201/eid2212.160805

**Published:** 2016-12

**Authors:** Gregory S. White, Kelly Symmes, Pu Sun, Ying Fang, Sandra Garcia, Cody Steiner, Kirk Smith, William K. Reisen, Lark L. Coffey

**Affiliations:** Coachella Valley Mosquito and Vector Control District, Indio, California, USA (G.S. White);; School of Veterinary Medicine, University of California, Davis, California (K. Symmes, P. Sun, Y. Fang, S. Garcia, C. Steiner, W.K. Reisen, L.L. Coffey);; Environmental Services Department, Maricopa County, Phoenix, Arizona, USA (K. Smith)

**Keywords:** St. Louis encephalitis virus, arbovirus, flavivirus, California, mosquitoborne virus, emerging, human pathogen, mosquito, bird, arbovirus surveillance, arbovirus sequencing, viral phylogenetics, global viral spread, sentinel bird, mosquito pool testing, viruses, vector-borne infections

## Abstract

St. Louis encephalitis virus infection was detected in summer 2015 in southern California after an 11-year absence, concomitant with an Arizona outbreak. Sequence comparisons showed close identity of California and Arizona isolates with 2005 Argentine isolates, suggesting introduction from South America and underscoring the value of continued arbovirus surveillance.

St. Louis encephalitis virus (SLEV; family *Flaviviridae*, genus *Flavivirus*) was recognized in California in 1937 and caused periodic epidemics in humans and equines until 1989, including a 1984 outbreak in Los Angeles (*1**–**3*). Even though US epidemics have not occurred since 1989, SLEV activity was documented continually in California until 2003, the year West Nile virus (WNV) activity was detected in the state. During 2003–2015, no SLEV activity was detected in California despite ongoing SLEV surveillance and a 6-fold statewide increase in mosquito pool testing in response to the invasion of WNV. The absence of SLEV activity suggested its elimination from California (*4**,**5*). 

In Arizona, SLEV has been detected less frequently than in California, with low enzootic activity reported most years during 1972–2006 (Arizona State Public Health Laboratory, unpub. data) and a single human case during 2010–2014 (*6*). In Maricopa County, which includes Phoenix, a human SLEV outbreak during July–October 2015 resulted in 23 confirmed cases and 1 death (Arizona State Public Health Laboratory, unpub. data). 

Beginning in July 2015, SLEV activity was detected in mosquito pools, and sentinel chicken seroconversions were detected in the Coachella Valley in Riverside County, California. Given the reemergence of SLEV in California and Arizona in summer 2015, the purpose of this study was to describe the temporal and spatial detection of SLEV in California, compare its circulation intensity with that of WNV in California in 2015, and define the genetic relatedness of SLEV from both states to SLEV from elsewhere to infer a possible origin and pattern of spread.

## The Study

**Figure 1:**
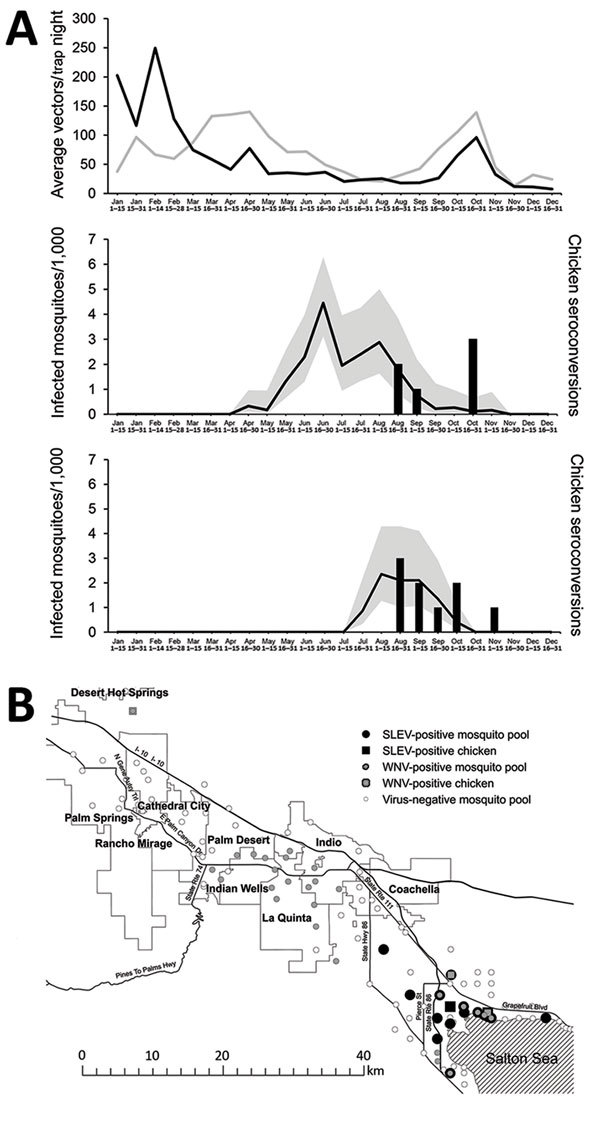
St. Louis encephalitis virus (SLEV) and West Nile virus (WNV) surveillance in mosquitoes and sentinel chickens in Coachella Valley, Riverside County, California, USA, 2015. A) Vector abundance (upper panel) from the same locations in all of Riverside County at bimonthly intervals during 2010–2014 (gray line) and in 2015 (black line), and infection rates for WNV (middle) and SLEV (lower) based on maximum likelihood estimates (black lines) with 95% CI (gray shading) in female *Culex tarsalis* and *Cx. quinquefasciatus* mosquitoes collected in CO_2_ and gravid traps and number of sentinel chicken seroconversions. B) Geographic locations of SLEV (black) and WNV (gray) activity identified by viral RNA detection in mosquito pools (circles) or sentinel chicken seroconversions (squares), July–October 2015.

Mosquito and arbovirus surveillance was conducted in the Coachella Valley in 2015 (Figure 1, panel A). SLEV RNA was first detected in a pool of *Culex tarsalis* mosquitoes by quantitative reverse transcription PCR on July 28, 2015, and subsequently in 37 more pools of the same species through October 6 (*7*). The number of SLEV-positive pools peaked at 23 during the first 2 weeks of August. WNV was detected in mosquitoes during April–November 2015, with a peak in the week of June 21. Although SLEV was detected only in *Cx. tarsalis* pools, WNV RNA was detected in 83 *Cx. quinquefasciatus* pools during April 24–November 5 and in 16 *Cx. tarsalis* pools during May 19–September 29. During the period of co-detection of both viruses (July–November), peak minimum infection rates were higher for WNV than for SLEV. 

Vector abundance did not parallel peak infection rates (June for WNV and August for SLEV). Instead, vector abundance in 2015 was lower at most times than the 5-year average, calculated as the geometric mean of female *Cx. tarsalis* and *Cx. quinquefasciatus* mosquitoes collected bimonthly in traps at the same locations during 2010–2014 (*8*). The trend of decreasing mosquito abundance in midsummer in southern California (especially in the Coachella Valley), concurrent with increasing arbovirus activity, has been well documented (*9*). This trend most likely relates to changes in age structure, with progressively more parous female mosquitoes tested as overall vector population numbers decline. Sentinel chicken seroconversion to SLEV, detected by enzyme immunoassay and confirmed by plaque-reduction neutralization test, was detected during August 28–November 9, with a total of 9 seroconversions in 104 chickens (8.7% seropositive) (*10*,*11*). WNV seropositive chicken serum samples were also reported starting on August 28, but with fewer (n = 6 [5.7%] of serum specimens tested) seroconversions to WNV than to SLEV.

Although most mosquito pools contained detectable RNA for only WNV or SLEV, 4 pools tested positive for both viruses. Both viruses were circulating in the summer of 2015 at the north and west shores of the Salton Sea in the Coachella Valley (Figure 1, panel B). SLEV activity was more focal than WNV activity and was limited to *Cx. tarsalis* mosquitoes collected in a 20-km radius near wetlands and agricultural habitats by the Salton Sea, whereas WNV activity spanned >80 km and was concentrated in *Cx. quinquefasciatus* mosquitoes collected in more densely populated residential habitats in the central part of the Coachella Valley. Co-circulation of both viruses in ecologically diverse habitats near the Salton Sea at the same time shows that early-season WNV activity did not preclude later SLEV circulation.

**Figure 2:**
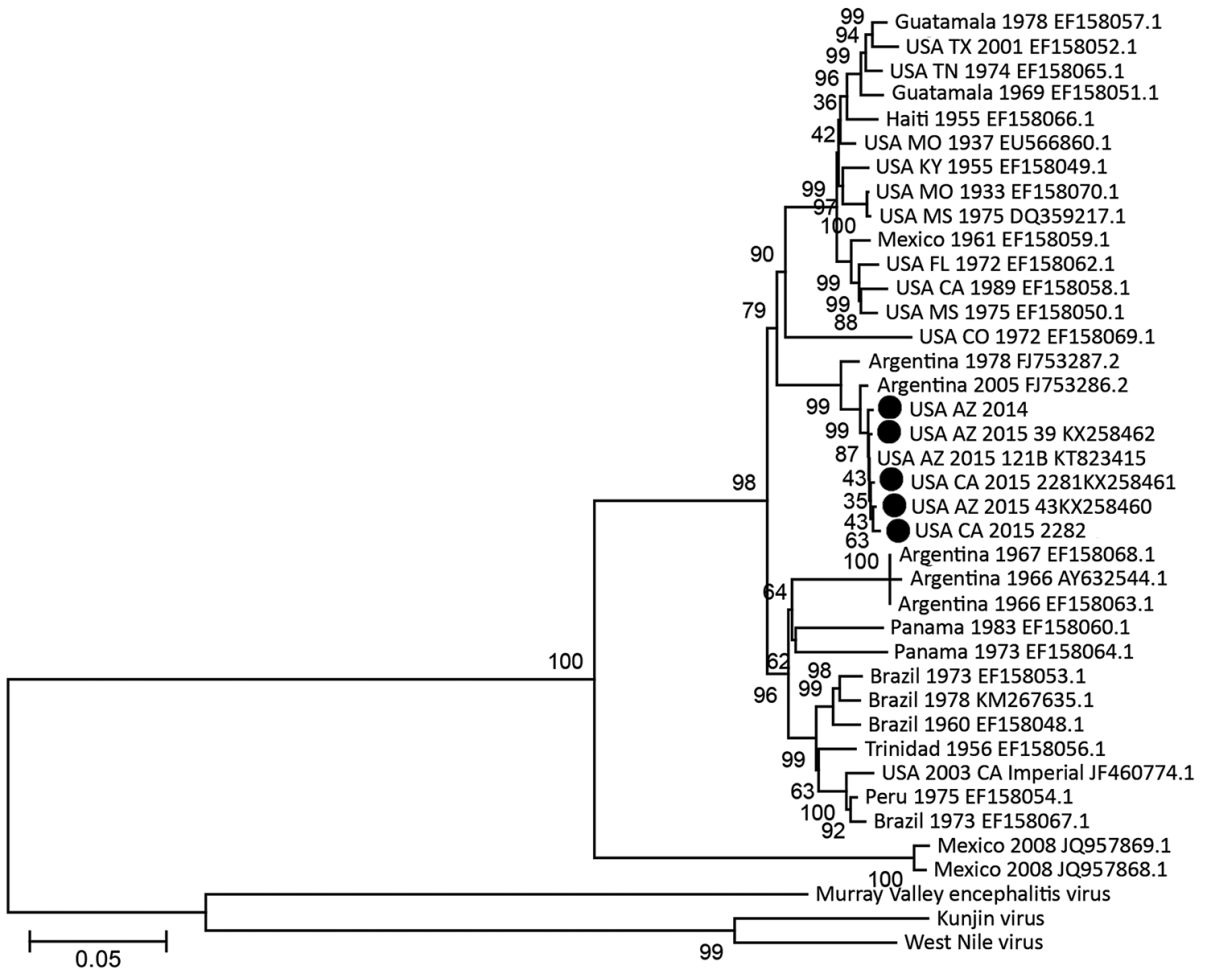
St. Louis encephalitis virus phylogeny with 2015 California (USA CA) and 2014 and 2015 Arizona (USA AZ) genomes (black circles). Complete nucleotide genomes (except for isolate 2282, which included only the E gene) were compared by using a neighbor-joining algorithm and 1,000 bootstrap replicates (support numbers at nodes) by using MEGA 7 (*14*). Isolates are named according to location, year of isolation, strain name for 2014 and 2015 isolates, and GenBank accession number. Scale bar indicates nucleotide substitutions per site.

Unlike California, where continual SLEV testing has been conducted since 1969, the absence of SLEV activity in mosquitoes in Arizona during 2010–2014 may have been due to a lack of recent SLEV testing. Maricopa County began testing mosquito pools for SLEV RNA during the 2015 human epidemic and then retrospectively detected SLEV in an archived WNV-positive mosquito pool from November 2014. We sequenced complete genomes of 1 SLEV isolate from California in 2015 and two isolates from Arizona in 2015, and partial genomes of the 2014 isolate from Arizona and 1 additional 2015 California isolate from reverse transcription PCR amplicons using SLEV primers (Table; GenBank accession nos. KX258460–62 [California], KX965720 [Arizona]). We further determined the phylogenetic relationships of the sequenced isolates with each other and with complete SLEV genomes from GenBank, including another 2015 Arizona isolate (strain 121B, GenBank accession no. KT823415) sequenced by the Centers for Disease Control and Prevention (Figure 2). The 2014 and 2015 California and Arizona SLEV isolates share >99% nucleotide identity with each other and also with their closest published relative, isolated from *Cx. quinquefasciatus* mosquitoes collected in Cordoba, Argentina, in 2005. The 2015 SLEV isolates are genetically distinct from the 2003 Imperial Valley California strain that was isolated before the 11-year absence of SLEV activity in the state. These results suggest that there was likely a single introduction of SLEV into the United States from South America (possibly Argentina) no later than November 2014, the earliest dated sample from which SLEV was isolated in Arizona, and that the virus spread in the summer of 2015 from Arizona to California. Notably, 1,710 mosquito pools representing 65,287 individual mosquitoes from the Coachella Valley were negative for SLEV during 2014.

**Table:** Primers used to sequence complete genomes of SLEV in study of reemergence of the virus in Arizona and California, USA, 2015*

Name	Sequence, 5′ → 3′	Location of primer binding site at 5′ nucleotide on SLEV 2005 isolate CbAr4005†
2300F	GGATTACACAGGGACTACTTGG	2339
2300R	TCTGTATGCTCTCCCACATTAAG	2672
2700F	CCTGAAGAAGCTGGAAGATGAG	2786
2700R	CGCTTTCAATAACGCCATCAC	3175
5700F	GGTGATTCAGCTAAACAGGAAGA	5753
5700R	GTGATTGCCATGGGTCCATTA	5936
800F	CAATCCTGGATATGCCCTAGTT	852
800R	ACGGTCCACAACATCTCTTT	1241
9000F	CCAAAGTTCTGGGAAATGGTTG	8981
9000R	CATAGGAATTCTCACGGCTCAT	9189
F1	GAGCGGAGAGGAAACAGATTT	17
F10	CGGAGCTGTGACTCTTGATTT	4976
F11	AGGCCGTATTGGGAGAAATC	5981
F12	CACGACGCAGTATGTGAACT	7099
F13	GGAGTGGACGTGTTCCATAA	8069
F15	GAGTGAACACCATGCCAAATC	9099
F16	TGGTAGGAGGAGTGCTGTAA	10393
F2	GGAGAAGTCATGGCTGGTAAA	1584
F3	CCCTGGAGTGAAGGAGAAATAAC	3276
F4	GGGTTCCCAACTACCAAGTTTA	5431
F7	GGTTGAGTGGCTAAGGAAGAA	9635
F8	CATTCTTGGCGGGTTTGTTC	3796
F9	GCAATAGCTGGGCTGATGTA	4315
R1	CGCTGGTCGCTAGAAAGATTAG	2488
R10	CGGAGCTGTGACTCTTGATTT	5418
R12	CAGATAGCCCTGCTTCCTTTAG	9099
R2	AGCACACAAGATGGGAAGAG	3985
R3	GAAGCTGGTGATCCACTCATAC	5651
R4	ACGATTCCGTCTTTCCTGTATG	7761
R5	GCCCACTCCTGTTCTGTTTATC	8417
R6	CATCCTGCTCCTGGTGAAAT	9924
R7	CCTGTCTTTCCAGGTGTCAATA	3185
R8	GGGATTGACCGTAACCAATCT	2023

*SLEV, St. Louis encephalitis virus. †GenBank accession no. FJ753286.2.

Because human SLEV viremia levels are low and insufficient to infect mosquitoes, the virus may have been introduced into the United States from South or Central America by a viremic migratory bird or possibly by an infected mosquito exploiting human transportation (*12*). Earlier US SLEV strains from Tennessee and Texas isolated in 1974 and 2001, respectively, are most closely related to 1969 and 1978 Guatemalan strains (Figure 2). A similar ancestral topology of Brazil and Peru strains from the 1970s to the 2003 California isolate also suggests movement from South to North America. The Salton Sea and associated habitats possess diverse avifauna and may serve as a resting point for SLEV-infected migratory birds that traverse the Pacific flyway (*13*). Alternately, SLEV may have come from elsewhere in the United States after being introduced from South or Central America, but sequences from US states reporting SLEV activity in recent years are not publicly available. In the case of east-to-west movement across the United States, postnesting birds may have mediated spread by way of agricultural areas of northern Mexico. However, it is unknown whether SLEV is active in Mexico or the Imperial Valley, which lies between Phoenix and the Coachella Valley, because surveillance is not performed in those regions.

Our findings highlight how mosquitoborne viruses are emerging and reemerging to establish autochthonous transmission, including SLEV in southern California that produced severe and fatal human disease in 2015 in Arizona (*6*). Prospective surveillance can identify viruses circulating in mosquitoes even in the absence of human cases of infection, as in Coachella Valley in 2015, and may provide an early warning of future outbreaks.
